# Supplementary material for: Targeting ADAR stability: a novel approach to combat glioblastoma
Source: Front Oncol. 2026 Aug 6;16:1827793. doi: 10.3389/fonc.2026.1827793 (PMC13489870; doi:10.3389/fonc.2026.1827793)
Supplement: Supplementary file 1 [file SupplementaryFile1.zip › Supplementary file 1/marker_genes_cor.pdf]

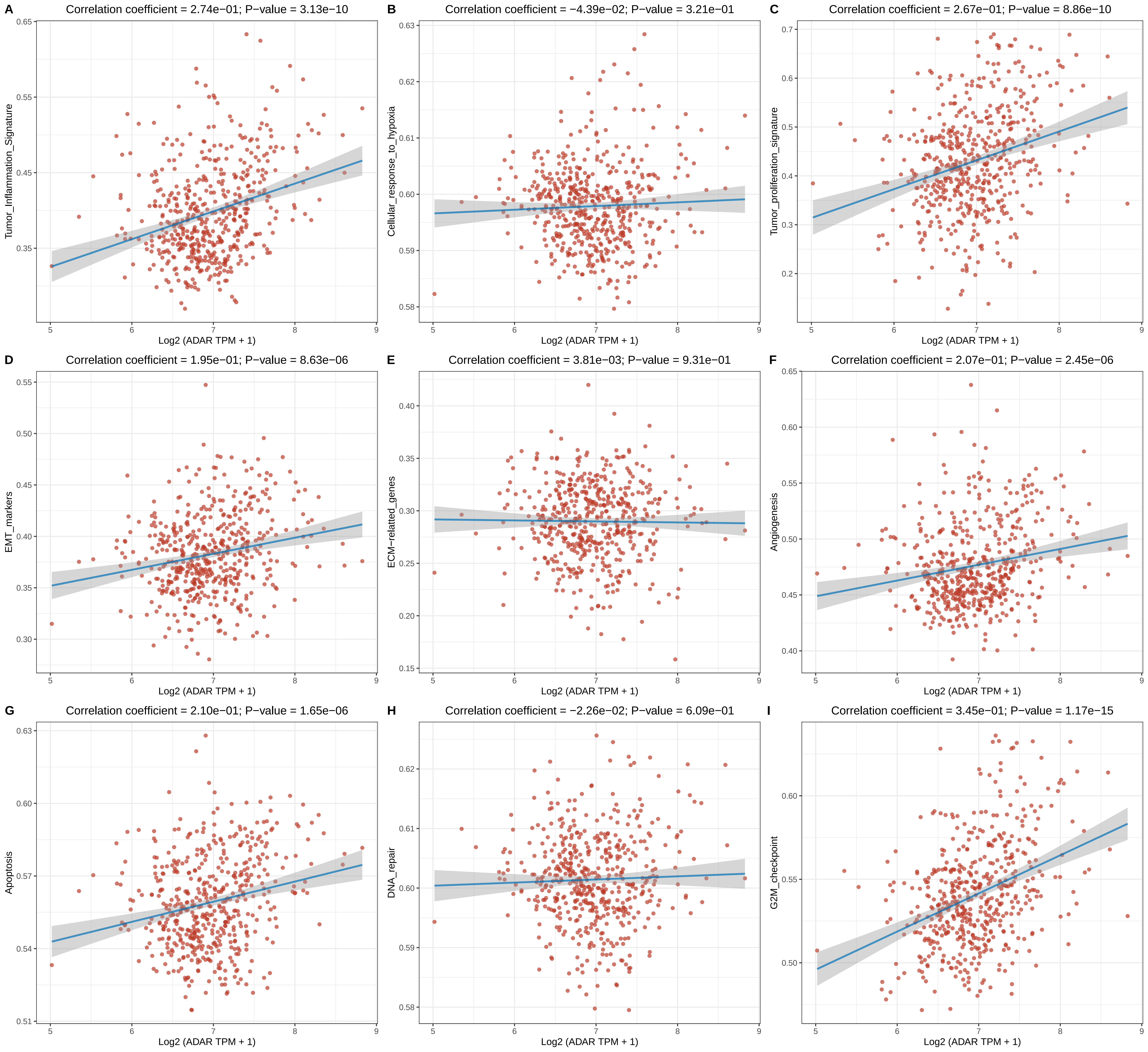
A
Correlation coefficient = 2.74e−01; P−value = 3.13e−10
Tumor_Inflammation_Signature
0.65
0.55
0.45
0.35
5
6
7
8
9
Log2 (ADAR TPM + 1)
B
Correlation coefficient = −4.39e−02; P−value = 3.21e−01
Cellular_response_to_hypoxia
0.63
0.62
0.61
0.60
0.59
0.58
5
6
7
8
9
Log2 (ADAR TPM + 1)
C
Correlation coefficient = 2.67e−01; P−value = 8.86e−10
Tumor_proliferation_signature
0.7
0.6
0.5
0.4
0.3
0.2
5
6
7
8
9
Log2 (ADAR TPM + 1)
D
Correlation coefficient = 1.95e−01; P−value = 8.63e−06
EMT_markers
0.55
0.50
0.45
0.40
0.35
0.30
5
6
7
8
9
Log2 (ADAR TPM + 1)
E
Correlation coefficient = 3.81e−03; P−value = 9.31e−01
ECM−related_genes
0.40
0.35
0.30
0.25
0.20
0.15
5
6
7
8
9
Log2 (ADAR TPM + 1)
F
Correlation coefficient = 2.07e−01; P−value = 2.45e−06
Angiogenesis
0.65
0.60
0.55
0.50
0.45
0.40
5
6
7
8
9
Log2 (ADAR TPM + 1)
G
Correlation coefficient = 2.10e−01; P−value = 1.65e−06
Apoptosis
0.63
0.60
0.57
0.54
0.51
5
6
7
8
9
Log2 (ADAR TPM + 1)
H
Correlation coefficient = −2.26e−02; P−value = 6.09e−01
DNA_repair
0.62
0.61
0.60
0.59
0.58
5
6
7
8
9
Log2 (ADAR TPM + 1)
I
Correlation coefficient = 3.45e−01; P−value = 1.17e−15
G2M_checkpoint
0.60
0.55
0.50
5
6
7
8
9
Log2 (ADAR TPM + 1)
